# A novel carbon tipped single micro-optrode for combined optogenetics and electrophysiology

**DOI:** 10.1371/journal.pone.0193836

**Published:** 2018-03-07

**Authors:** Dénes Budai, Attila D. Vizvári, Zsolt K. Bali, Balázs Márki, Lili V. Nagy, Zoltán Kónya, Dániel Madarász, Nóra Henn-Mike, Csaba Varga, István Hernádi

**Affiliations:** 1 Kation Scientific LLC, Minneapolis, Minnesota, United States of America; 2 Szentágothai Research Center and Center for Neuroscience, University of Pécs, Hungary; 3 Department of Experimental Neurobiology, University of Pécs, Hungary; 4 Department of Applied and Environmental Chemistry, University of Szeged, Hungary; 5 NAP-B Entorhinal Microcircuits Research Group, Department of Physiology, University of Pécs, Hungary; University of Pennsylvania, UNITED STATES

## Abstract

Optical microelectrodes (optrodes) are used in neuroscience to transmit light into the brain of a genetically modified animal to evoke and record electrical activity from light-sensitive neurons. Our novel micro-optrode solution integrates a light-transmitting 125 micrometer optical fiber and a 9 micrometer carbon monofilament to form an electrical lead element, which is contained in a borosilicate glass sheathing coaxial arrangement ending with a micrometer-sized carbon tip. This novel unit design is stiff and slender enough to be used for targeting deep brain areas, and may cause less tissue damage compared with previous models. The center-positioned carbon fiber is less prone to light-induced artifacts than side-lit metal microelectrodes previously presented. The carbon tip is capable of not only recording electrical signals of neuronal origin but can also provide valuable surface area for electron transfer, which is essential in electrochemical (voltammetry, amperometry) or microbiosensor applications. We present details of design and manufacture as well as operational examples of the newly developed single micro-optrode, which includes assessments of 1) carbon tip length–impedance relationship, 2) light transmission capabilities, 3) photoelectric artifacts in carbon fibers, 4) responses to dopamine using fast-scan cyclic voltammetry *in vivo*, and 5) optogenetic stimulation and spike or local field potential recording from the rat brain transfected with channelrhodopsin-2. With this work, we demonstrate that our novel carbon tipped single micro-optrode may open up new avenues for use in optogenetic stimulation when needing to be combined with extracellular recording, electrochemical, or microbiosensor measurements performed on a millisecond basis.

## Introduction

Optogenetics is the combination of genetic and optical methods that can be used to induce or inhibit well-defined events in specific cells of living animal tissue [1, 2]. Electrophysiological studies are one of the most popular methods used in neuroscience research whereby microelectrodes are used to record action potentials (spikes) from a single neuron or local field potentials (LFPs) in brains of live animals. The combination of light delivering optical and electrical recording elements in one single device used to detect light-induced neuronal responses is referred to as an optical electrode (optrode) [3].

Because of its simplicity and high temporal resolution, the earliest, groundbreaking optrode solution [4] of attaching an optical fiber to a conventional extracellular metal microelectrode has been extensively used over the past 10 years [5–10]. These single-channel optrodes targeting individual or small groups of neurons typically in restrained or anesthetized animals have been mostly bifurcated or fork-like structures. Optetrodes comprising of four metal microwire bundles arranged around an optical fiber and cut to extend 300–1,000 μm beyond the end of the optical fiber could also permit a multichannel readout for optogenetic control in freely moving mice [11]. Due to their tip structure, some of these optrode designs may cause substantial tissue damage when inserted into deep brain target areas. Moreover, a metal microelectrode that is unequally illuminated compared with a reference electrode may be susceptible to light-evoked electrical artifacts or the Becquerel effect [12–15] thereby deflecting recorded LFPs or other low-frequency biopotentials.

Nevertheless, development of the coaxial single micro-optrode design has, for the most part, removed these limitations. As a novel approach, light was locally delivered through the aperture at the tip of a tapered, metal layered optical fiber to nearby neurons. The sharp tip has been observed to allow easy tissue penetration while causing minimal damage, whereas simultaneous neuronal activities can be recorded through the thermally metalized gold tip of the optrode [16]. As such, this approach has been further advanced by placing the tapered optical fiber in a double barrel micropipette and pulled so that the tip of this microprobe consists of a cleaved optical fiber and hollow core, *i*.*e*., a fillable micropipette. The latter can be filled with electrolyte solution and serve as a recording barrel. An aluminum coating is evaporated on the probe to act as both an optical reflector and electrical conductor to prevent optical losses through the tapered region and to allow electrical recording [17, 18].

In another original optrode innovation, others have used an approach using four sharpened graded-index optical fibers coupled with a center-positioned tungsten wire microelectrode tightly bound with one another and integrally coated with a smooth thin layer of glass. This coaxial design satisfies the structural requirements needed for use in deep brain structures in large-bodied animals while simultaneously limiting damage to tissue during penetration, and allowing for multimodal functions such as light delivery, extracellular recordings, and fluorescence detection [19]. A new class of flexible neural probes (single optrodes) fabricated from polymer, metal, and composite materials have been successfully developed for use in simultaneous optogenetic stimulation, neural recording, and drug delivery in freely moving mice [20, 21].

The goal of this paper is to describe the manufacturing, properties, and *in vivo* performances of our newly developed carbon tipped single micro-optrode. For classification of optrodes, see recent reviews [3, 22, 23]. In the present observations, favorable aspects of the coaxial design were combined with the strength and multimodal capabilities of carbon fiber (CF) used in extracellular recordings and electrochemistry or microbiosensor applications. Carbon fiber monofilaments demonstrate outstanding physicochemical properties and serve as excellent base electrodes on a micrometer scale. In microbiosensors, the carbon tip is covered with biological sensing elements such as an enzyme, receptor protein, antibody, or nucleic acid immobilized in a conducting polymer matrix [24].

## Materials and methods

### Fabrication of carbon tipped single micro-optrodes

Optrode blanks were built from three major components: (1) a 9 μm diameter individual carbon fiber (pitch-type, F500, Donacarbo, Japan) and (2) a 125 μm diameter optical fiber (FG105LVA, core diameter: 105 μm, Thorlabs), whereby components of both numbers 1 and 2 were suctioned by mild vacuum into a (3) 1.5 mm × 100 mm (outer diameter × length) borosilicate glass capillary tubing (B150-86-10, Sutter Instruments, USA). The 1 m long optical fiber had an FC/PC connector on one end and was jacketed with a protective 0.8 mm diameter PVC furcation tubing on its entire length. The CF was previously attached with electrically conductive epoxy glue to a fine-gauge silver wire, which was soldered onto a gold-plated industry-standard miniature pin (220-P02-100, Cooper Interconnect, USA) to provide a low-impedance connection for recording electrical signals. Prior to assembly, the acrylate coating of the optical fiber was stripped at a length of about 50 mm. The free end was ground at a right angle so that the final grinding was performed on a rotating disk bearing a 3M lapping film of 0.5 μm diamond particles (816–390, Ted Pella, USA). The two fibers were then fixed in the top end of the glass capillary by applying a small drop of standard epoxy glue. Completed blanks were laid to rest overnight.

Pulling was performed using a fully automated puller device developed in-house. The puller system was controlled with an NI6221 multifunction board placed in a desktop computer and programmed with LabVIEW (National Instruments, USA). Two ends of the blank were vertically fixed using holder clamps. The upper and lower clamps, as well as the spiraled heater (Kanthal) wire, were mounted on linear movements actuated by stepper motors. Timing and intensity of heating currents, application of vacuum and all linear movements were programmatically controlled (steps summarized in S1 Fig). This resulted in a 120 μm diameter thin glass rod, which extended beyond the end of the optical fiber to a length of about 7 mm and encapsulated the CF in the longitudinal axis. Excessive length of the borosilicate glass rod was removed by repeated dipping of the tip into concentrated hydrofluoric acid [25] to form an approximately 4 mm long miniature glass rod with a dome-shaped glass end. The protruding carbon fiber was then etched into a sharp conical tip at the required lengths under a microscope using high voltage sparks (S2 Fig) [26, 27]. Some of these glass tips were also ground on a 0.5 μm lapping film to form a glass disk around the disk-shaped CF ending. Lastly, the upper end of the pulled optrode was glued into a 3D-printed (Ultimaker2, Ultimaker, The Netherlands) ABS plastic encasement, which held in place both the incoming optical fiber and the signal relaying gold-plated pin. The lower cylinder of the plastic part provided a convenient way to attach this optrode to the stereotactic manipulators.

### Scanning electron microscopy

Scanning electron microscopy (SEM) was carried out using an S-4700 field emission scanning electron microscope (Hitachi, Japan) operated by the Department of Applied and Environmental Chemistry, University of Szeged, Hungary. Electrode samples for electron microscopy were coated with conductive films of gold with the aid of a K650X sputter coater (Quorum Technologies, UK).

### Impedance and light power measurements

The impedance of the carbon fiber lead element was determined at 1 kHz using an ICM impedance conditioning module (FHC, USA). The instrument operated in constant voltage mode and the impedance of the electrode was calculated from the ratio between the driving voltage and the output voltage, in a voltage divider configuration. Measurements were carried out under a light microscope in a drop of physiological saline suspended in a 5 mm diameter platinum loop that served as a reference and counter electrode in a two-electrode configuration (S3 Fig).

Light power was measured in mW using a PM100USB power and energy meter interface equipped with an S151C sensor (used up to 20 mW) or with an S121C sensor for power levels above 20 mW (all from Thorlabs, USA). Light power projections of micro-optrode tips were measured in air using an ABS plastic adaptor attached to the sensor module to keep 2 mm distance between the light emitting tips being measured and the light sensing flat surface.

### Measuring photoelectric artifacts

Photoelectric or light-induced artifacts due to the Becquerel effect were measured in side-illuminated traditional glass-insulated tungsten microelectrodes (W1011, Kation Scientific, USA) placed in physiological saline and were compared with those detected in the present carbon micro-optrodes. For these measurements, a 2 W, 445 nm blue laser diode was used. Light was passed through 1 mm optical fiber (FT1000UMT), whereas the power of the projected light was calibrated using an S121C sensor (Thorlabs, USA). Tungsten microelectrodes, as well as optrodes with three different carbon tip lengths, were placed 0.5 mm apart from the end of the optical fiber. Light was turned on for 200 ms by a computer-controlled solid-state switch and potential measurements were performed against an unlit Ag/AgCl counter electrode using a BioAmp amplifier (Supertech, Hungary) using bandpass filter settings of 1.5 to 150 Hz. Recordings were digitized at 25 kHz and visualized using an NI6221 multifunction board via desktop computer and programmed with LabVIEW (National Instruments, USA).

In a second set of experiments using a similar approach, micro-optrodes with three different carbon tip lengths equal to 0 (ground), 25 μm, or 100 μm were tested for photoelectric artifacts. Different compared with the previous studies, carbon tips positioned in the longitudinal axis of the light conducting glass tip were exposed to light delivered via built-in 125 μm diameter optical fiber at 473 nm from a 70 mW diode-pumped solid-state laser source (Shanghai Laser & Optics Century, China). Light projection power was calibrated as described above.

### Calibration of voltammetric responses to dopamine of the carbon tip

The magnitude of responses of carbon microelectrodes in electrochemical applications is greatly dependent on the electroactive surface available for electron transfer. For this reason, voltammetric responses to DA of the carbon micro-optrodes were studied using 9 μm × 100 μm (diameter × length) carbon tips that provided significantly greater carbon surface than 25 μm tips.

Electrochemical responses of the 100 μm long CF tip aimed at increasing concentration of DA were calibrated at room temperature in a 1 mL acrylic glass flow cell. Constant 1 mL/min inflow of buffered saline (PBS; 8.2 mM Na2HPO4, 1.8 mM NaH2PO4, 138.9 mM NaCl, 4.4 mM KCl, pH 7.4) was fed by gravity through a bottom inlet port, whereas outgoing fluid was drained on top using a peristaltic pump. Dopamine (Tocris, UK) was freshly dissolved in PBS and injected into the inflow stream through an injection valve to produce test analyte concentrations ranging from 15.6 to 1000 nM in doubling concentrations. Carbon tips were positioned 1.5 mm deep in the inlet port and responses to DA were measured using fast-scan cyclic voltammetry (FSCV) at a 400 V/s scanning rate within the range of –0.4 to 1.3 V against a Ag/AgCl reference electrode. Scans were repeated at 10 times/s and the resulting traces were recorded using a Chem-Clamp potentiostat equipped with a 1 MΩ head-stage (Dagan, USA). The system was controlled using two multifunction data acquisition cards (PCI-6221 and PCI-6711, both from National Instruments, USA) via a desktop computer. Acquired and digitized voltammetric data were analyzed using Demon Voltammetry and Analysis Software (DVAS) written in LabView (National Instruments, USA) and obtained from Wake Forest University [28]. Peak oxidation currents were selected and used for further calculations. Limit of detection was determined using linear regression analysis and calculated as follows: 3 × SD(b)/a, where SD(b) is the standard deviation of the intercept, and a is the slope according to the ordinary least-squares estimation of the linear regression. The slope of the regression line was considered as the sensitivity of the microelectrodes. In order to determine area specific responsiveness, surface areas were calculated by a cone on a cylinder approach and dimensions were determined using SEM and light microscopic images.

### Ethics statement

This study was carried out in strict accordance with the recommendations in the decree no. 40/2013 (II. 14.) of the Hungarian Government and guidelines of the EU directive, 2010/63/EU. All aspects of this protocol were approved by the Animal Welfare Committee of the University of Pécs and by the National Scientific Ethical Committee on Animal Research of the Hungarian Government’s Ministry of Food and Agriculture (permit number: BA02/200/1/2015). All efforts were made to minimize the number of animals used and prevent or ameliorate their suffering.

### Animal handling and viral transfection

Male Wistar rats (Toxi-Coop, Hungary) weighing 440 to 540 g were used in all experiments. Rats were housed in the animal facility of the University of Pécs, Hungary under standard conditions at a constant temperature of 22 ± 2 °C with food and water supplied *ad libitum*. Upon completion of the experiments, animals were killed with excessive amounts of general anesthetics (ketamine) and were perfused transcardially with paraformaldehyde fixative for histological studies.

For viral infections, rats were deeply anesthetized with 5% isoflurane and craniotomy was performed in a stereotaxic frame above the target area (lateral: 2.5 mm, anteroposterior: –4 mm relative to Bregma). Channelrhodopsin-2 expression was induced by administration of a viral solution of AAV5.CaMKIIa.hChR2(H134R)-eYFP.WPRE.hGH (Addgene 26969P, 2.31 × 10^11^ GC/mL, Penn Vector Core, USA) and injected unilaterally into the dorsal hippocampus. A 50 nL aliquot was delivered by slow pressure injection from an 80 μm tip sized micropipette over 5 minutes. Following the end of injection, the micropipette was left to rest in place for an additional 15 minutes and was then slowly retracted. Finally, the craniotomy wound was treated and animals were given suitable postoperative care. Recording sessions commenced 2–4 weeks following viral injection.

### *In vivo* optogenetic stimulation and extracellular recordings

For recording sessions, rats were initially anesthetized with a combination of ketamine (100 mg/kg, CP-Pharma, Hungary) and diazepam (20 mg/kg, Gedeon Richter, Hungary) and administered intraperitoneally. Animals were mounted using a stereotaxic frame and stable anesthesia was maintained throughout the experiment with additional low doses of ketamine administered as needed. Optogenetic light stimulation and extracellular recordings were achieved by means of our novel carbon tipped, coaxial single micro-optrodes method presented above. As such, the connector end of the optical fiber was attached to the light source and the optrode was mounted in the stereotactic manipulator. Optrodes with 25 μm carbon tips were lowered into the target area of the brain using a suitable micromanipulator. Successful light-induced neuronal recordings were taken from the stereotactic coordinates of –3.5 to –5.3 mm anteroposterior and 1.5 to 3.5 mm medio-lateral from the Bregma. Light stimuli were delivered via built-in optical fiber at 473 nm from a 70 mW diode-pumped solid-state laser source (Shanghai Laser & Optics Century, China) using continuous stimulation epochs. Electrophysiological signals were recorded through the carbon tips and amplified using two simultaneously activated amplifiers; one for spike recordings with bandpass filter settings at 300 to 6,000 Hz (Neurolog, Digitimer, UK) and one for recording LFPs with bandpass filter settings ranging from 1 to 150 Hz (BioAmp, Supertech, Hungary). The amplified signals were digitized at 25 kHz with 16-bit resolution using CED Power 1401 A/D converters and the Spike2 v6.07 software (Cambridge Electronic Design, UK). Channelrhodopsin/eYFP expression was histologically documented with a Zeiss LSM 780 confocal microscope (Zeiss, Germany).

### *In vivo* voltammetry

Optrodes with 100 μm carbon tips were applied as working microelectrodes in in vivo voltammetric measurements for DA detection in brains of anesthetized rats. Anesthesia and stereotaxic procedures were performed as described above. Three holes were drilled in the skull; one for the optrode serving as the working electrode, one for the Ag/AgCl reference electrode, and one for the stainless-steel wire twisted-pair bipolar stimulating electrode (PlasticsOne, USA). The working electrode was stereotaxically positioned above the nucleus accumbens, whereas the stimulating electrode was positioned above the ventral tegmental area (VTA) of the midbrain. The reference electrode was attached to the surface of the dura mater above the cortex of the hemisphere opposite the working electrode. Voltammetry was conducted by means of a Dagan Chem-Clamp potentiostat (Dagan, USA) in connection with the DVAS and National Instruments’ hardware and LabView software as described above. Bipolar electrical stimulation in the VTA was performed using a Model 2200 stimulus isolator (A-M Systems, USA) with parameters as follows: 50 biphasic pulses at 50 Hz, 4 ms in length, and with ±500 μA amplitude for each stimulation trial. Both the working and stimulating microelectrodes were carefully lowered stepwise into the target areas of the brain while stimulation trials and FSCV measurements were repeated at each step. After establishing a stable (control) detection of peak oxidation currents in response to electrical stimulation, 5 mg/kg norepinephrine-DA reuptake inhibitor, nomifensine (Sigma-Aldrich, Germany), was injected intraperitoneally to enhance and validate increase of oxidation currents within the nucleus accumbens. Electrical stimulations and voltammetric measurements were repeated every 3 minutes following administration of nomifensine.

### Statistical analysis

Significant differences between raw data sets were performed using one-way analysis of variance (ANOVA) with Tukey’s range test for post hoc analysis. Data are presented as means ± SD of the number (n) of observations. Two-tailed significance was determined using a P value of <0.05. All curve fittings and statistical analyses were performed using OriginPro 8 (OriginLab, USA).

## Results

### Design of the single micro-optrode

**Fig 1:**
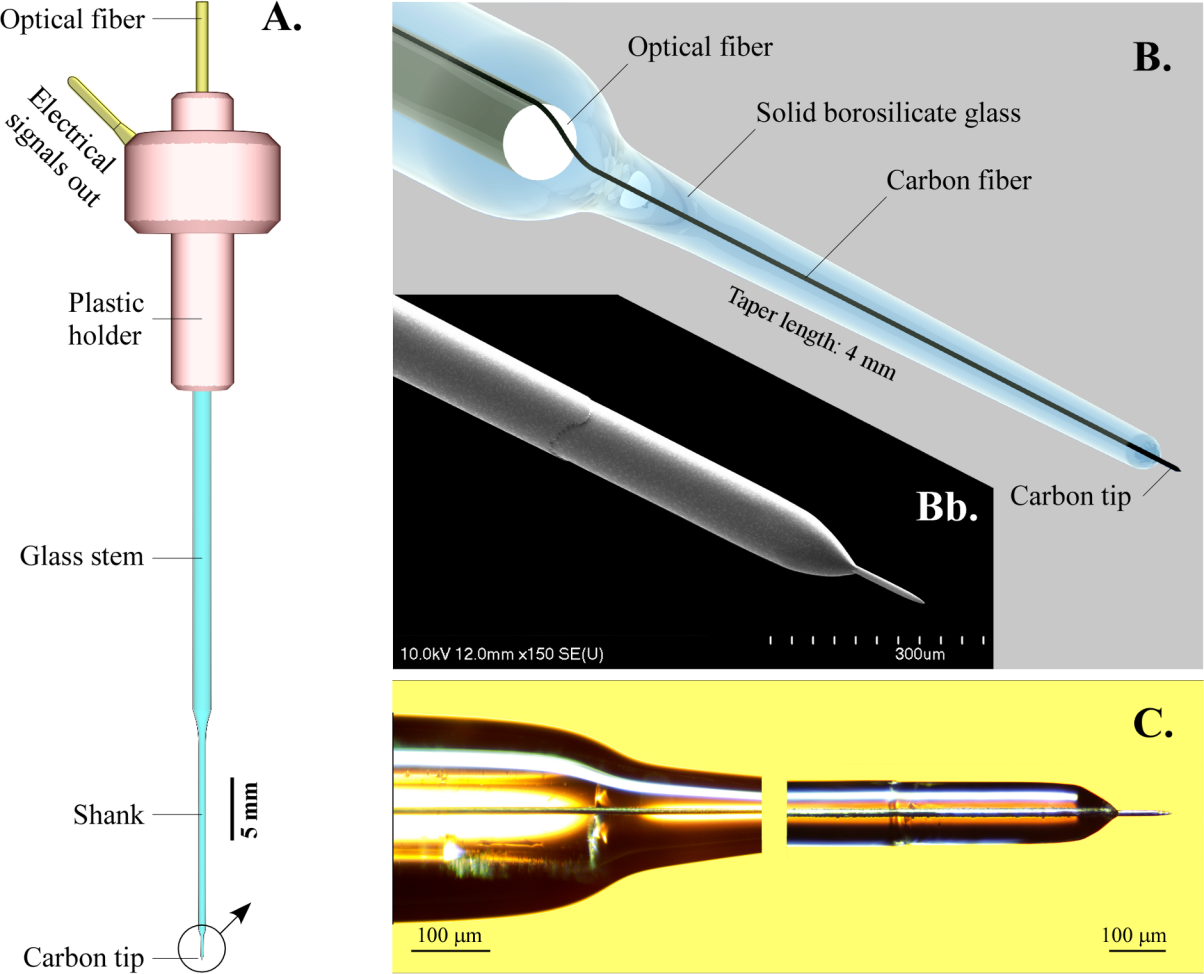
The carbon tipped coaxial single micro-optrode. (A) Overall view illustrating major components. Light was delivered through the optical fiber and conducted further by the borosilicate glass extension light guide. Electrical signals picked up by the carbon tip were transmitted to amplifiers by the gold-plated miniature pin built in the plastic holder. (B) A three-dimensional rendering illustrating the construction of the apex. The solid glass light guide also served as a mechanical support and tightly sealing electrical insulator. (Bb) Scanning electron microscopic image of the final glass taper segment with a protruding 100 μm long sharpened carbon tip. (C) Light microscopic image of the optical fiber end, the light guiding glass taper and center-positioned carbon fiber extending from the dome-shaped glass ending.

The single micro-optrode presented in this study was designed as a combination of a new type of CF microelectrode supplemented with a coaxially positioned optical fiber for light delivery (Fig 1). The PVC-jacketed 125 μm optical fiber having an FC/PC connector on its free end provided a reliable means to deliver light from its source to the optrode. On its top end, the CF recording element was attached to a miniature gold-plated pin using conductive epoxy glue to provide electrical connection to the carbon tip below. The top assembly was finally encapsulated in a 3D-printed ABS plastic holder mounted onto the end of the glass capillary as shown in Fig 1A. All parts here were fixed together with standard epoxy glue. The structure of the tip of the micro-optrode is shown in Fig 1B and 1C.

**Fig 2:**
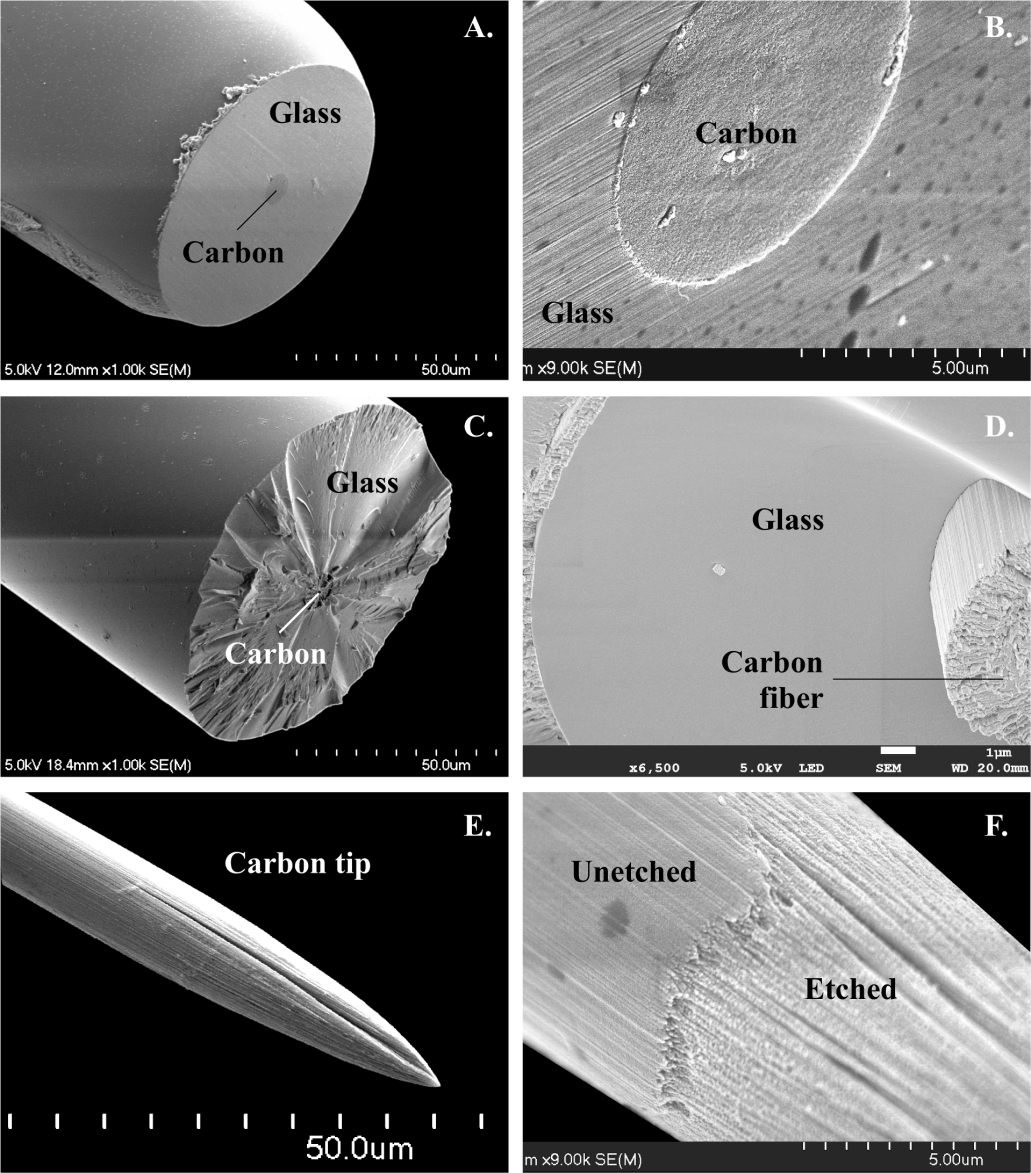
Scanning electron micrographs showing tip structure of micro-optrodes. (A, B) View of a ground tip demonstrating the center position carbon disk surrounded by an annulus of glass. The glass served as mechanical support, an electrical insulator, and a light guide. (C, D) Endings of intentionally broken tips exposing tight junctions between carbon fiber and glass sheathings. (E, F) The surface roughness of a carbon tip revealing the effects of spark etching on a pitch-type carbon fiber. Note the longitudinal bundles of carbon fiber. See also panel D.

Details of pulling the optical and CF into borosilicate glass sheathings are given in S1 Fig. Pulling with vacuum inside caused the glass capillary to enclose tightly both fibers in a 0.4 mm diameter, 20 mm long, sturdy shank (Fig 1A). In the resulting micro-optrode light was projected through the end of the optical fiber that was ground perpendicularly to the longitudinal axis and guided further down by the overextending solid borosilicate glass light guide containing the carbon fiber in its axis and tapered over a length of 4 mm then terminated in a 120 μm diameter (Fig 1A and 1B). Two types of apexes were formed: (a) a dome-shaped end with various lengths of protruding carbon tips, or (b) the tip was ground perpendicularly to the axis. In the latter configuration, the electrically conductive 9 μm carbon disk was surrounded by a light-projecting glass annulus (Fig 2A and 2B). The length of the carbon tip protruding from the glass end was carefully set under a light microscope using controlled spark etching (S2 Fig). This method produced a sharp conical tip at the end of the CF with highly reproducible lengths. The 25 μm long tips were optimized for electrophysiological recordings, whereas the 100 μm long tips were primarily made for electrochemical applications. Tip quality was checked visually under a light microscope along with the impedance measurements (S3 Fig). The overall yield of successfully completed carbon optrodes was 80% of the starting number of blanks.

### Impedance of carbon tips

Our newly developed pulling method resulted in a unique structure. The lead element CF was embedded in the center of a tapered solid glass rod (Figs 1 and 2) shaped by controlled-speed pull, programmed heating current and timed application of vacuum so that a tight seal was formed between the softened glass and carbon fiber. This seal was tested with distilled water or physiological saline under a light microscope where a change in the refraction of light could be easily detected in the presence of even the thinnest water layer that may have been driven up by capillary force. Only those with no water leak were selected for further processing. In addition, the junction between the two materials was studied by means of SEM, as shown in Fig 2. Scanning electron microscopic images of intentionally broken glass rod tapers in Fig 2C and 2D illustrate the tight junction between glass and CF.

**Fig 3:**
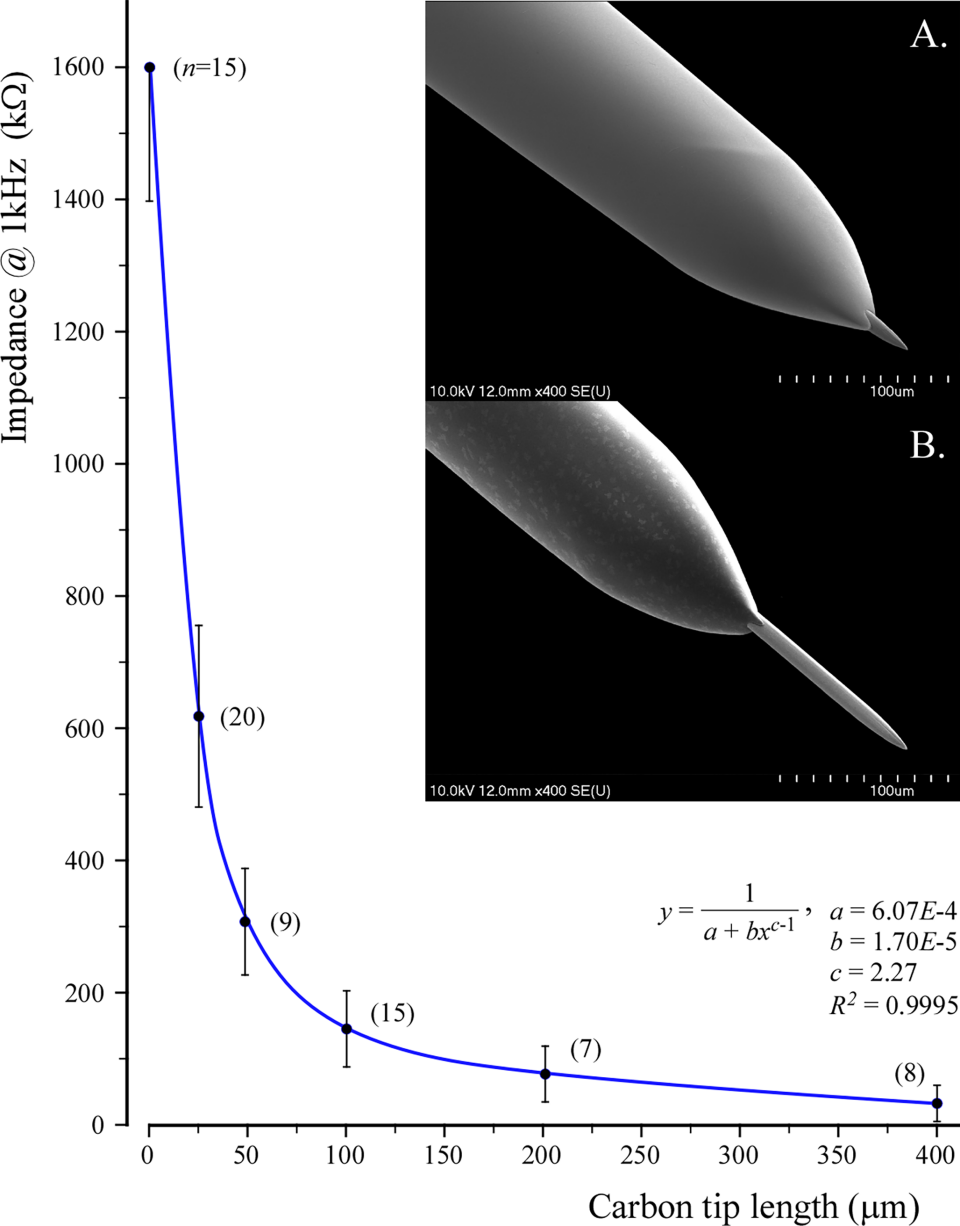
Impedance of the carbon tip as a function of length. The curve was best fitted with an extended Langmuir adsorption isotherm equation. Mean impedances ± SD of *n* carbon tips for each length were determined in saline at 1 kHz and plotted against lengths as shown. Impedances of ground-tip carbon disks were also determined and their lengths were taken as 1 μm. Scanning electron micrographs of 25 μm and 100 μm long carbon tips are shown as insets A and B, respectively.

Impedance measurements using sine waves at 1 kHz in physiological saline (S3 Fig) demonstrated a strong power decay-type relationship between lengths of the protruding CF tips and their impedances (Fig 3). The curve was fit and described via an extended Langmuir adsorption isotherm equation as shown. The relationship was described by the equation *y* = 1/(6.07 × 10^−4^ + 1.70 × 10^−5^ *x*^2.27–1^), where y is the impedance in kΩ measured at 1 kHz, and *x* is the length of carbon fiber tips in μm. The close to unity goodness of fit, *R*^*2*^ = 0.9995, suggested a strong, constant relationship between tip lengths and their impedances.

### Light transmission and photoelectric artifacts

**Fig 4:**
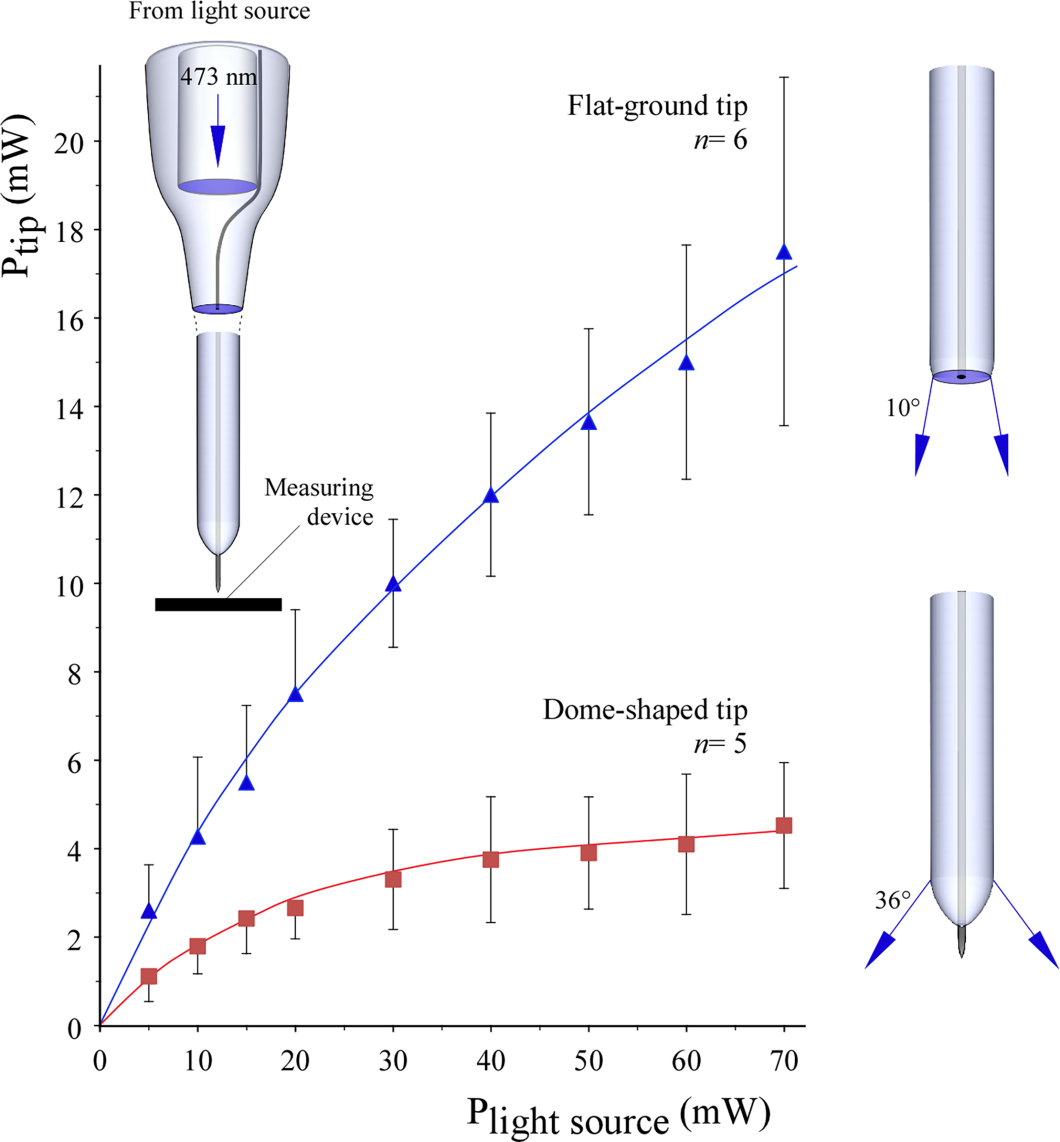
Light projection power through the tip as a function of light source power. Light was delivered through the built-in optical fiber connecting to the 473 nm laser diode light source. Light power projected by optrode tips and detected with a flat sensor surface in air were measured using ground or dome-shaped tips. Mean ± SD of *n* micro-optrodes were calculated and shown.

Measured at its output connector, our diode-pumped solid-state laser source output a maximum of 70 mW light power at 473 nm. Equipped with a 1 m long, 125 μm diameter optical fiber, a 25 ± 1.4 mW (mean ± SD of five fibers) the maximum was detected at the other, perpendicularly ground bare end. Light transmission through the tip of the completed micro-optrode was measured in both dome-shaped and flat-ground apices as a function of the power of the light source (Fig 4). Using a flat measuring surface placed 2 mm away from the glass endings in air, ground-tip optrodes (Fig 2) were capable of transmitting 17.4 ± 3.9 mW (mean ± SD, *n* = 6) light power at 70 mW source power. Under the same conditions, but in the setting of dome-shaped tips (Fig 3A and 3B), significantly less light power was measured at all source power levels (Fig 4). For example, 4.5 ± 0.3 mW (mean ± SD, *n* = 5) light power was projected by the dome-shaped tips at 70 mW source power. The most likely explanation for this difference may be the more focused light projection by the ground endings to the sensor surface as compared with the dome-shaped ones. Estimated half angles of light projections were 10° and 36° for ground and dome-shaped glass tips, respectively. Given the 120 μm diameter of the glass light guide, flat-ground tips projected 1539 mW mm^–2^ while dome-shaped ones 398 mW mm^–2^ in the forward direction using a 70 mW light source.

**Fig 5:**
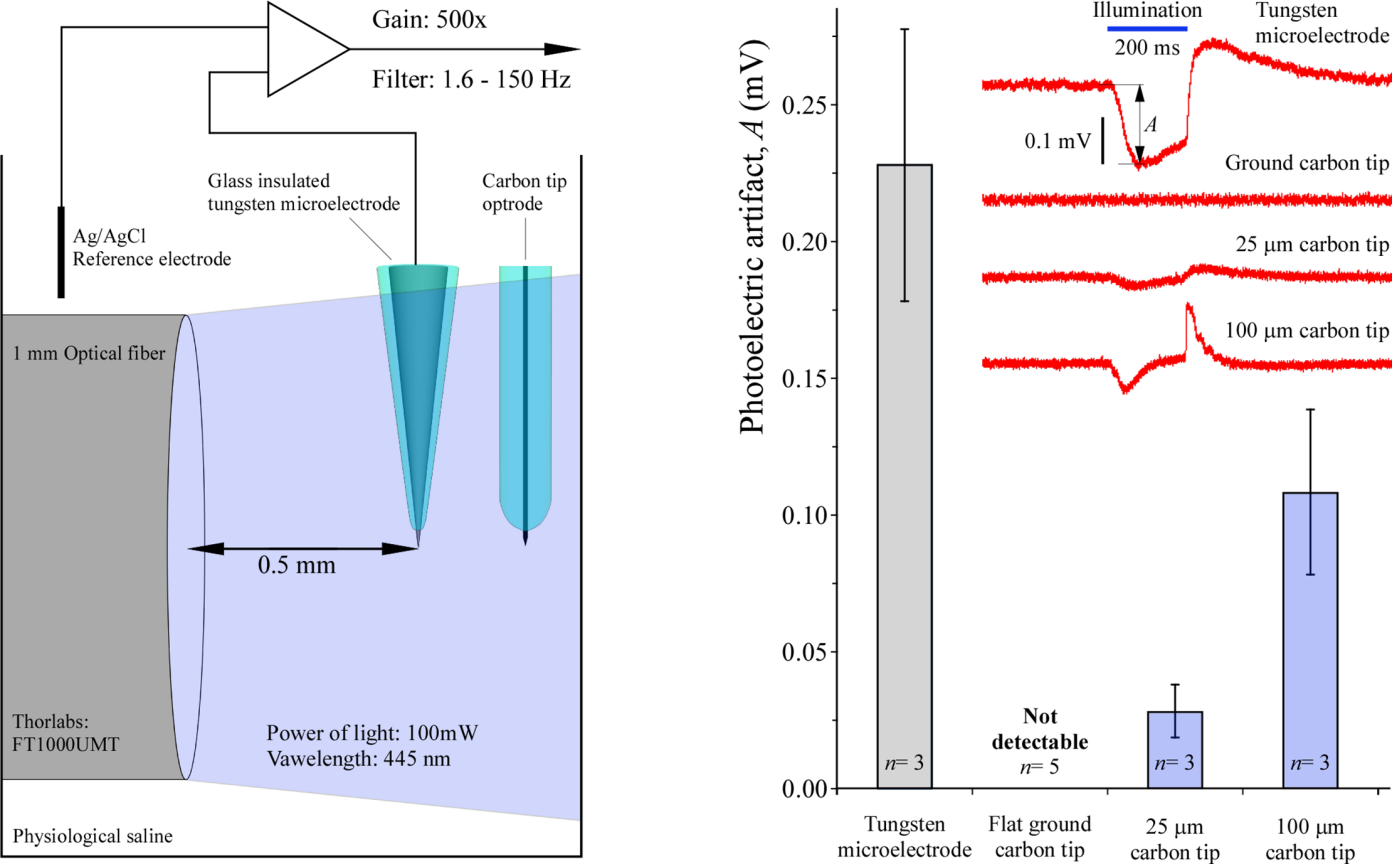
Photoelectric effects in tungsten and carbon tip microelectrodes. Light-induced potentials were recorded from laterally illuminated microelectrodes as illustrated on the left. Amplitudes (*A*) of the initial phase of the artifacts were measured as shown by the sample recordings at the top of the right panel. Data are presented as mean ± SD. All comparisons were significant (*P* < 0.01).

Lateral illumination of tips was performed using a 1 mm diameter optical fiber that transmitted 100 mW light power at 445 nm (Fig 5). Measured in physiological saline against an unlit Ag/AgCl counter electrode, initial amplitudes of the photoelectric artifacts in tungsten microelectrodes were significantly greater, 0.228 ± 0.048 mV (mean ± SD, *n* = 3, *P* <0.01 by ANOVA), than in any versions of the carbon fiber micro-optrodes. Under the same conditions, a clear relationship was observed between the lengths of exposed (uninsulated) carbon tip and the magnitude of their light-induced artifacts. In order of lengths, 100 μm and 25 μm long carbon tips produced 0.108 ± 0.030 mV (*n* = 3) and 0.028 ± 0.007 mV (*n* = 3) photoelectric artifacts, respectively. No artifacts were detected in any of the five tested flat-ground-tip carbon micro-optrodes.

**Fig 6:**
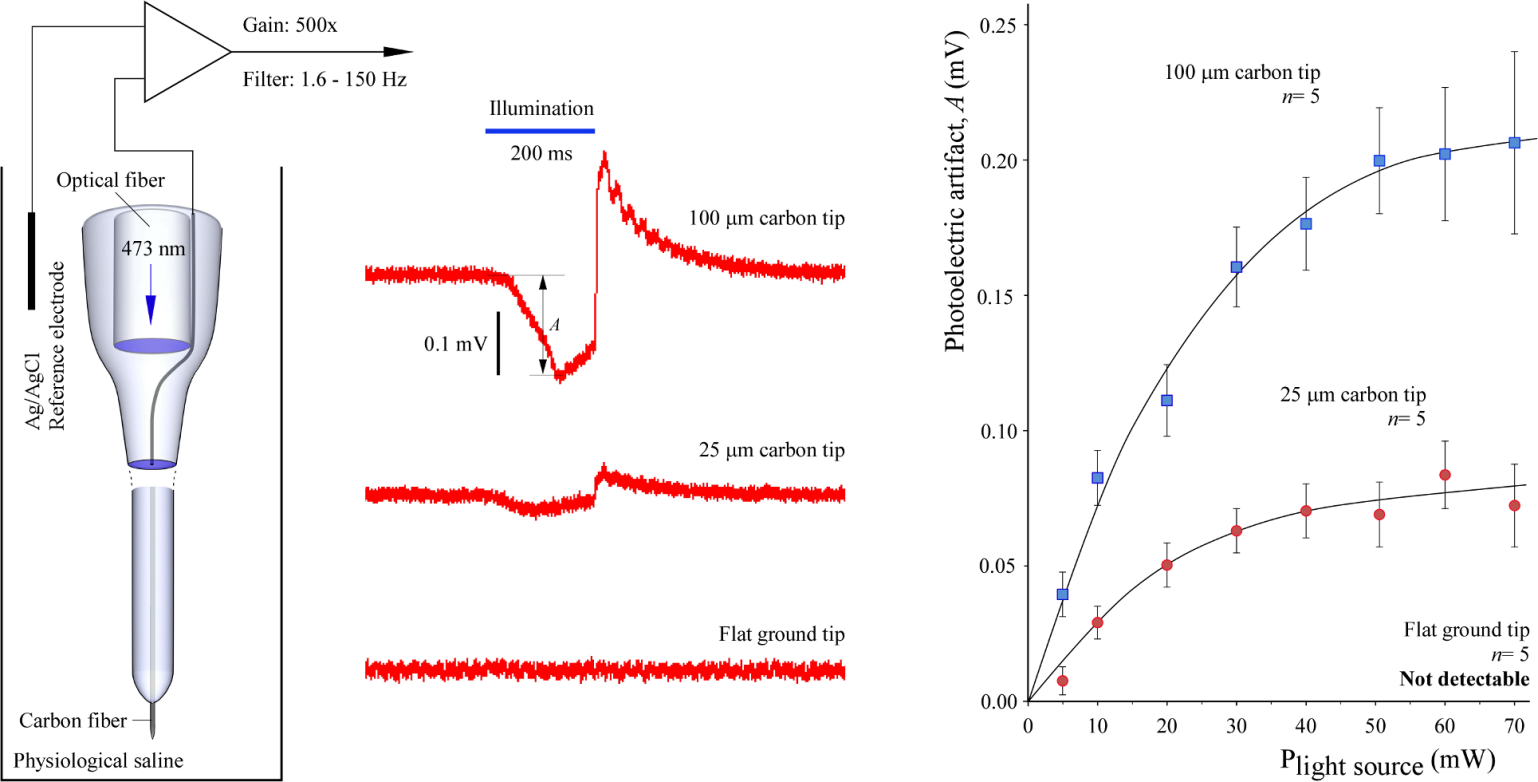
Photoelectric effects in carbon tipped micro-optrodes. Upon illumination through the built-in optical fiber, the photoelectric artifacts in the carbon fiber were dependent on light power and the lengths of the uninsulated carbon tips. Recordings were made as shown on the left and amplitudes (A) of the initial phase of the artifacts were measured as indicated on the sample traces in the middle. Data are presented as mean ± SD. Note the complete lack of artifacts in the ground-tip micro-optrodes.

In the next set of experiments, photoelectric artifacts were induced using the 125 μm optical fiber as a source of light built in the carbon fiber micro-optrodes (Fig 6). The lead element 9 μm carbon fiber was integrated into the longitudinal axis of the borosilicate glass support and light guide extended over the ending of the optical fiber. Amplitudes of the initial phase of the artifacts in three lengths of exposed, uninsulated, and in contact with the physiological saline, carbon tips were studied as a function of the intensity of light output at 473 nm by the laser diode light source. A strong relationship was observed between length of exposed carbon tip and amplitude of light-induced artifacts. For example, at maximum (70 mW) illumination the 100 μm long carbon tips produced almost three times greater artifacts than the tips 25 μm in length. Numerical values were 0.206 ± 0.033 mV (mean ± SD, *n* = 5) and 0.072 ± 0.015 mV (*n* = 5), respectively. Similar to lateral lighting (Fig 5), no artifacts were recorded in any of the five tested flat-ground-tip carbon micro-optrodes. It should be noted that 70 mW light power at the source produced about 4 to 18 mW at the tip of the carbon micro-optrodes depending on the geometry of the tip (Fig 4).

### Optogenetic stimulation and *in vivo* recordings

**Fig 7:**
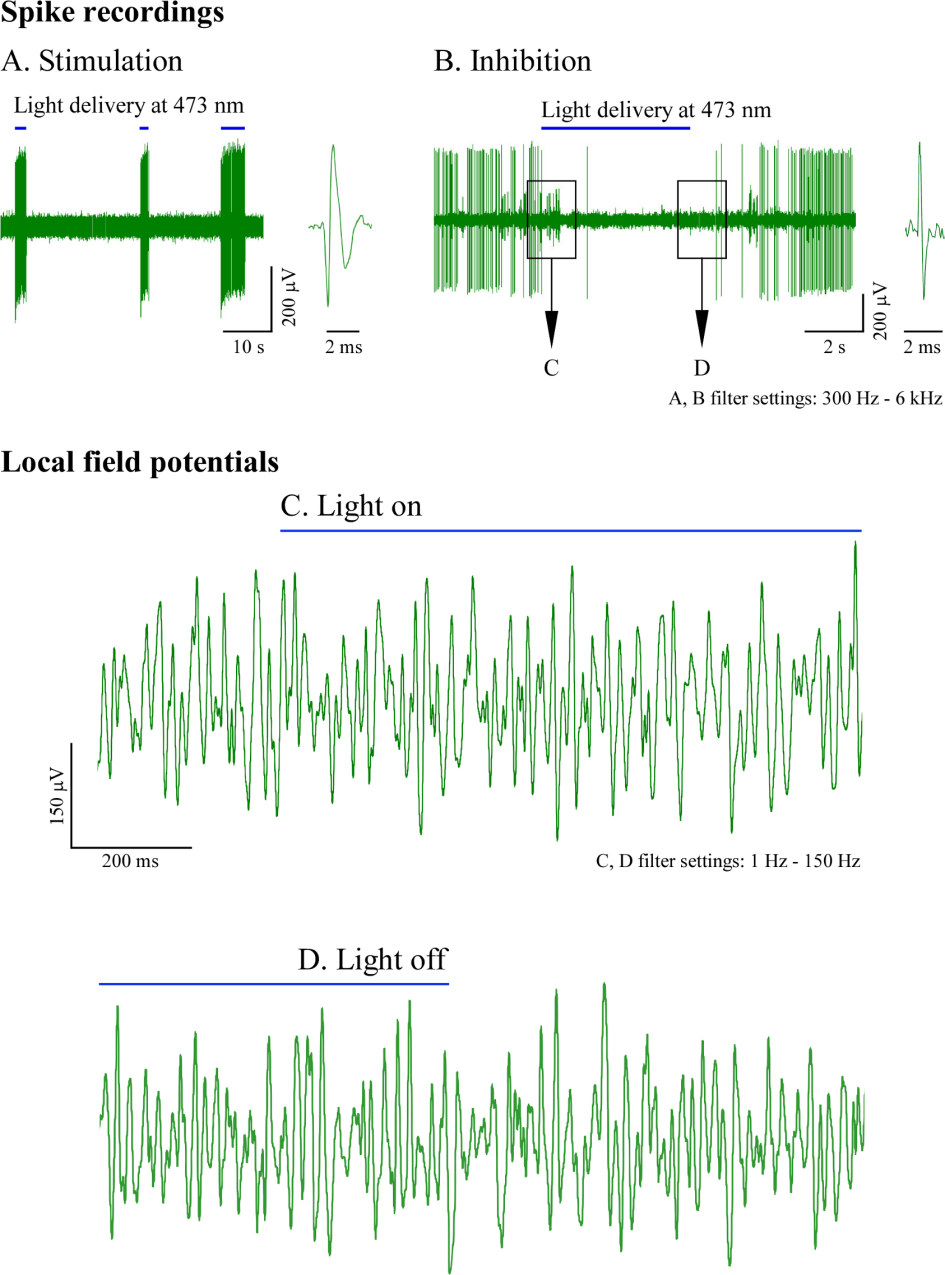
Optogenetic stimulation and extracellular recordings with a 25 μm carbon tip. Sample recordings were taken from the hippocampus of the rat brain transfected with channelrhodopsin-2 using a dome-shaped glass tip for light projection with a 25 μm carbon tip as recording element. Light stimuli were delivered at 473 nm from a 70 mW laser source in continuous epochs, as shown by horizontal bars. (A, B) Light-sensitive neurons were either excited or, in a smaller number, inhibited during delivery of light. (C, D) Local field potentials were recorded with filter settings that ranged from 1 Hz to 150 Hz. Segments designated in panel B were detailed showing the recorded traces around the onset (C) and conclusion (D) of the light stimulation. No noticeable photoelectric artifacts were recorded under these conditions.

*In vivo* performance of the micro-optrode was studied in the laboratory rat transfected with channelrhodopsin-2 and immobilized in a stereotactic frame under deep anesthesia. Before these experiments, light projection from a dome-shaped tip using the 473 nm laser source was visualized in agar-agar gel as shown in S4A Fig. The power of light transmitted through the tips of the optrodes was previously quantified and shown in Fig 4. Single unit recordings were taken from 26 neurons during the course of seven insertions in the brains of three transfected rats using two micro-optrodes with 25 μm carbon tip protruded from the dome-shaped glass tip (for picture, see Fig 3A). The optrode was carefully lowered into the hippocampus while exploratory light stimuli were released at 70 mW source power. Neurons in various layers of dorsal hippocampus responded to light stimulation particularly well in a respective dorsoventral range of 2.0 mm to 3.5 mm from the brain surface. These neurons in the target area also displayed remarkable fluorescent signal in postmortem histological visualization confirming the successful transfection with the channelrhodopsin-containing viral vector (S4B Fig). Of the 26 recorded cells, 13 were stimulated by the impact of light, whereas three of them ceased firing in response. Representative neuronal spike and LFP recordings in response to optogenetic stimulation are shown in Fig 7. No noticeable photoelectric artifacts were seen during LFP recordings.

Recording sessions lasted for no longer than three hours. Impedances of the recording 25 μm carbon tips were determined just before tissue penetration and after completion of the experiment following a vigorous wash in distilled water. The pre-recording impedance was 620 ± 92 kΩ (mean ± SD, *n* = 2) measured at 1 kHz and this value did not change significantly as compared to the post-recording impedances implying the lack of leakage of tissue fluid in between carbon fiber and its insulating glass sheathings.

### Voltammetric responses of carbon tips to DA

**Fig 8:**
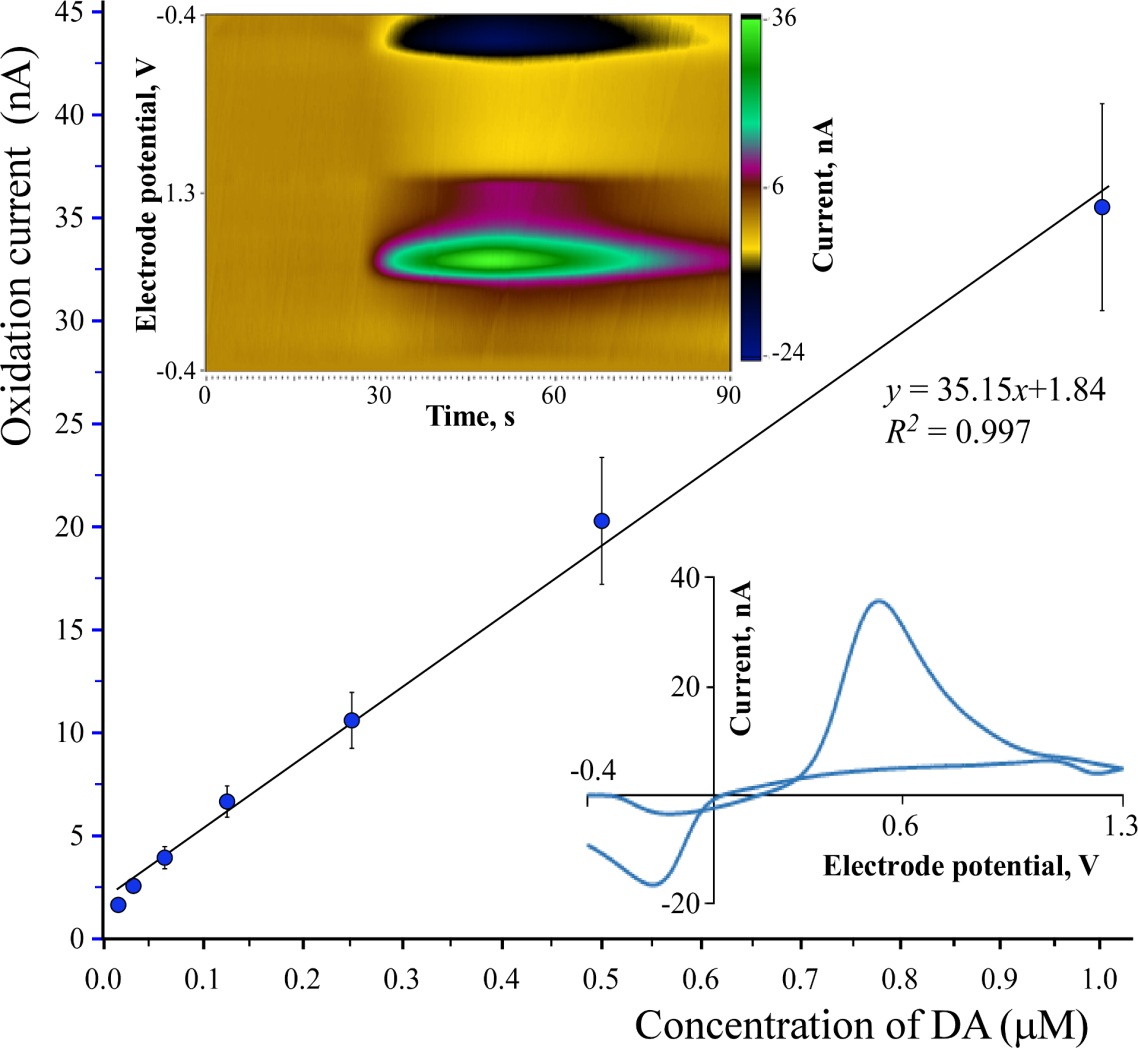
Voltammetric responses of carbon tips to DA. Average peak fast-scan cyclic voltammetry oxidation currents plotted against the concentration of dopamine (DA) dissolved in phosphate-buffered saline. Values represent the mean ± SD of six micro-optrodes with 100 μm carbon tips. (Top left inset) Visualization of DA-evoked oxidation currents as a function of the electrode potential and time using the Demon Voltammetry and Analysis Software. (Bottom right inset) A representative background-subtracted cyclic voltammogram recorded at 1 μM DA concentration.

Fast-scan cyclic voltammetry was performed in a flow cell at a scan rate of 400 V/s against a Ag/AgCl reference electrode in PBS containing freshly dissolved DA. Calibration curves in six individual 100 μm carbon tip optrodes (for example, see Fig 3B) were constructed based on the peak oxidation currents in DA solutions with concentrations ranging from 15.6 nM to 1000 nM and applied in a doubling manner. To see the variability between carbon tips, mean responses ± SD were calculated at each DA concentration. Results are summarized in Fig 8. A linear regression analysis resulted in the equation *y* = 35.15*x* + 1.84 with *R*^*2*^ = 0.998. Hence, the average sensitivity of the entire carbon tip to DA in the six optrodes was 35.15 ± 4.95 nA/μM DA (mean ± SD, *n* = 6) with a calculated limit of detection of 29.1 nM DA. Percent coefficient of variation between tips was 14%, showing the good reproducibility of the complex manufacturing technology. The surface area available for electron transfer was 2850 μm^2^ so the corresponding area specific responsiveness was 12.3 ± 1.7 pA/μM/μm^2^ (mean ± SD, *n* = 6).

### *In vivo* voltammetry

**Fig 9:**
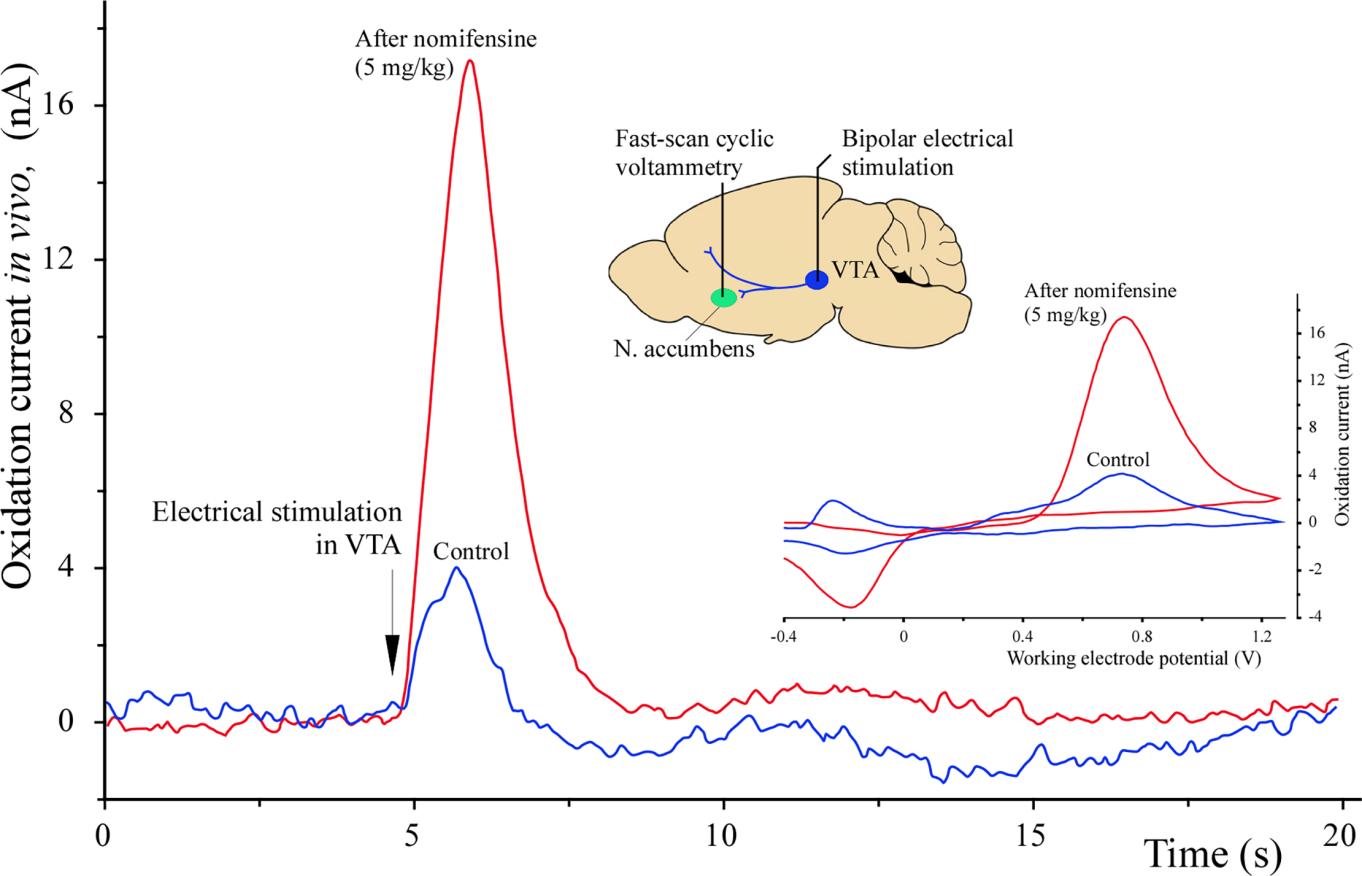
Stimulation-evoked oxidation currents in the nucleus accumbens. A representative experiment exemplifying the performance of a micro-optrode with 100 μm carbon tip in fast-scan cyclic voltammetric (FSCV) determination of peak oxidation currents in the nucleus accumbens in response to electrical stimulation of the ventral tegmental area (VTA). The prenomifensine peak current was taken as control. The maximum oxidation current was measured 21 minutes postnomifensine intraperitoneal administration. The inset in the middle shows the sites of electrical stimulation and FSCV recordings in a silhouette of the rat brain. Traces of actual voltammograms are shown in the lower right inset.

This sample experiment was carried out in the brain of an optogenetically unmodified (wild-type) rat to exemplify the response *in vivo* of a micro-optrode equipped with a 100 μm long carbon tip. The micro-optrode was lowered into the nucleus accumbens whereas the bipolar stimulating microelectrode went into the VTA of the midbrain. First, the optimal sites for electrical stimulation and voltammetric recording were localized followed by the determination of the peak oxidation current in response to electrical stimulation. The results are summarized in Fig 9. Under these conditions and repeating the control measurement three times, the background-subtracted peak oxidation current by FSCV in the nucleus accumbens was 4.02 ± 0.26 nA (mean ± SD, *n* = 3) and this value was taken as control for the next trial. Next, nomifensine as a potent norepinephrine-DA reuptake inhibitor was intraperitoneally administered at a dose of 5 mg/kg and electrical stimulations along with the FSCV measurements were repeated every 3 minutes. In response to the systemically applied nomifensine, a significant, 4.4-fold increase was measured in the electrical stimulation-evoked peak oxidation currents, which reached its maximum at 21 min postnomifensine and produced 17.56 nA. Effects of nomifensine were gradually diminished to control level during about 90 min.

Just before the *in vivo* application, one of the six carbon micro-optrodes tested previously for DA responses was recalibrated in DA solution producing an equation of *y* = 32.36*x* + 2.16 with *R*^*2*^ = 0.998 by linear regression. Comparing the *in vivo* measured peak oxidation currents to this calibration curve, and if all oxidative electron transfers were due to DA molecules, which is not necessarily the case, the pre- and postnomifensine currents would have signaled 57.5 nM and 475.9 nM DA levels respectively in the nucleus accumbens in response to the electrical stimulation of the VTA.

## Discussion

The present carbon tip single micro-optrode solution offers several improvements over previous solutions. The slender coaxial design and the sharply pointed tip of our approach allowed a smooth penetration into the rat brain as observed in our *in vivo* experiments. As for the tissue damage during insertion, no histological analysis of such was performed in this study. However, based on these data we suggest the following: A 120 μm diameter cylindrical object with micrometer-sized tip, *i*.*e*., the present micro-optrode, may self-evidently cause significantly less tissue damage than the earliest optrode construction of a blunt-end 200 μm diameter optical fiber glued to 150 μm diameter tungsten microelectrode with a distance of 400 to 500 μm between the two tips [4]. Similar single optrode designs have been in use to this date [5, 7, 8, 10]. Accordingly, due to reduced tissue damage, swelling, and/or bleeding associated with our slender coaxial micro-optrode, we hypothesize that transfected light-sensitive neurons may require less light power for optogenetic activation in single optrode experiments.

The early makers of CF microelectrodes could not find a constant relationship between impedance and carbon tip length [29] likely due to leakage of saline or tissue fluid in between CF and the surrounding glass wall driven by capillary force. In our experience, we also observed that a simple pull of CF into a glass-coated microelectrode did not provide a tight seal between the two components. Improvements to the CF–glass seal were later perfected by using polyester [30] or epoxy [31–33] sealants, polishing by heat [34], or pulling CF into a thickened glass sheathing [35]. However, unlike the case of glass-coated tungsten microelectrodes [36, 37], no obvious relationship between carbon tip length and impedance has been established to date. Our present method using a vacuum during the pull resulted in a tight seal between the carbon fiber and the surrounding glass light guide, which prevented any inward suction of electrolyte fluids by capillary forces. Because of the tight junction between carbon fiber and the embedding glass in our solution, a power decay-like equation was obtained by plotting impedances of the carbon tip protruding from the glass support against their lengths.

It has been shown that effective channelrhodopsin-2-evoked spiking of neurons requires an estimated minimal light power of 1 mW mm^−2^ [38–40]. Propagation of blue light in brain tissue is limited by scattering and absorption so that only 10% of the light intensity reaches the 1 mm tissue depth measured from the light providing device [38]. Light flux density at the glass tips of our novel micro-optrodes is greatly dependent on the geometry of its ending. Measured in the forward direction in air by a flat surface detector some 30 to 80% of light power projected from the built-in optical fiber was lost. The flat-ground tips projected 1539 mW mm^−2^ whereas dome-shaped ones produced 398 mW mm^−2^. The difference might be due to the differing half angles of projections, which were 10° and 36° for ground and dome-shaped tips, respectively. However, even the dome-shaped micro-optrodes were proved to provide adequate illumination for inducing optogenetic changes in channelrhodopsin-2 transfected light-sensitive neurons in our *in vivo* sample experiments.

We have shown that under the same conditions, carbon tips of our micro-optrodes are significantly less prone to generate photoelectric artifacts than tips of traditional tungsten microelectrodes. This is an important finding raising the possibility of a more extensive usage of carbon materials in various types of optrodes to decrease photoelectric artifacts when needed and possible. Amplitudes and time kinetics of the light-induced voltage deflections in tungsten tips in our experiments are in agreements to those of published by Han *et al*., 2009 [13]. These artifacts have been repeatedly reported in light-exposed metal electrodes used in various experimental conditions [12, 15, 41, 42]. Corresponding to the Becquerel effect, a strong dependence of photoelectric artifact on the lengths of the exposed carbon tips that is in contact with the surrounding electrolyte. This suggests a need for using the shortest possible carbon tips when low frequency biopotentials are recorded.

In the present solution, the capabilities of carbon microelectrodes in electrophysiological recordings and their capabilities in electrochemical or micro-biosensor applications [24] were completed with an integrated optical fiber for *in vivo* optogenetic experiments. We have shown that the newly-developed optrodes were quite capable of optogenetic stimulation and recordings of spikes or LFPs from light sensitized hippocampal neurons of the rat brain. Most of the recorded light-responsive neurons were excited and increased their firing rate upon light delivery whereas a few of them ceased firing in response to light [39] in our validating sample experiment. No significant photoelectric artifacts were seen in LFP traces when recordings were taken using a 25 μm carbon tips.

Based on previous studies, carbon tips are very well suited for electrochemical applications where magnitude of an electrical response is greatly dependent on the surface area available for electron transfers. The greater the carbon surface area the greater their response to an electroactive analyte and better the signal to noise ratio. For this reason, we used optrodes with 100 μm long carbon tips that provided 2850 μm^2^ surface and performed well in FSCV detection of DA. A 14% coefficient of variation in response to DA was found between these carbon tips which represented a good reproducibility for the complex manufacturing technology. It should also be noted that many previous studies using carbon fibers for electrochemical measurements mostly neglected to calculate the exact electroactive surface areas that were available for electron transfers. The technology we present here allowed production of micro-optrodes with carbon tips of good geometrical reproducibility. Nevertheless, the simple geometrical calculation of surface area may not be adequate as the roughness of carbon surface shown by our SEM images may increase the area available for electron transfer further complicating parameters of adsorption and desorption of analyte molecules. The *in vivo* DA concentrations detected in our experiments due to the enhanced release of DA in the nucleus accumbens evoked by electrical stimulation of the VTA in the absence or presence of nomifensine corresponded well to values reported by previous studies [43–45]. It should also be noted that higher DA concentrations were measured in freely-moving animals than in anesthetized ones [44–46].

In summary, our novel carbons tipped single micro-optrode represents major improvements over previous solutions in its class. The co-axial, slender and highly pointed design may cause a minimal possible tissue damage during insertion in the brain. The lead element CF integrated in the center axis of the tapering glass light guide extending beyond the light delivering optical fiber is significantly less prone to generating photoelectric artifacts than metal microelectrodes. This construction delivers adequate amounts of light power to perform optogenetic stimulation and records spikes or LFPs from deep brain nuclei in good qualities in response. As an additional advantage, carbon tips of various lengths are capable of serving as working microelectrodes in electrochemical or possible future micro-biosensor applications.

## Supporting information

S1 Fig Steps of pulling carbon micro-optrodes. Blanks were made in 1.5 mm diameter borosilicate glass tubes containing optical and carbon fibers. The carbon fiber ended in a gold-plated pin for electrical connection. After vertically fixing the blank in the upper and lower clamps, the heater spiral was positioned so that the end of optical fiber was level with the upper edge of the spiral. Then the spiral was moved down by 15 mm. Once the glass was softened by electrical heating of the spiral, a vacuum was applied and the lower clamp was moved down by 25 mm at a speed of 2 mm/s. Following a 15 s cooling period, the heater spiral was positioned down by 3 mm; the clamp and the movement mechanism were disconnected using a latching device. Lastly, the glass was softened again with electric heating and the final tip was formed by free fall of the lower clamp. All other movements were actuated by lead screws connected to stepper motors as well as timing of events and applying heater currents were programmably executed using a computerized system.
(TIF)

S2 Fig Micro-machining of the carbon tip. Carbon tip lengths were finalized under a light microscope using spark etching as shown. High voltage electric discharges were generated between the carbon fiber and a sharp tungsten counter microelectrode resulting in a sharply pointed carbon tip.
(TIF)

S3 Fig Quality control and measurement of carbon tip impedance. Impedance of micromanufactured carbon tips was determined under a light microscope in a drop of applied physiological saline. Occurring at the same time, tips were visually inspected for imperfections and checked for possible inward liquid suction by capillary forces between the carbon fiber and glass support.
(TIF)

S4 Fig Visualization of light projection and expression of channelrhodopsin-2 in the target area. (A) The scattering of light leaving a dome-shaped tip was visualized in agar-agar gel. The borosilicate glass taper extended beyond the edge of the integrated optical fiber and served as a light guide. The half angle of most of the forward light projection was estimated at about 36°. (B) Histological section from the hippocampal region of the rat brain displaying the channelrhodopsin-2 expression. The *in vivo* recordings were taken from the same area.
(TIF)
