# Supplementary figures and images for: A novel carbon tipped single micro-optrode for combined optogenetics and electrophysiology
Source: PLoS One. 2018 Mar 7;13(3):e0193836. doi: 10.1371/journal.pone.0193836 (PMC5841794; doi:10.1371/journal.pone.0193836)

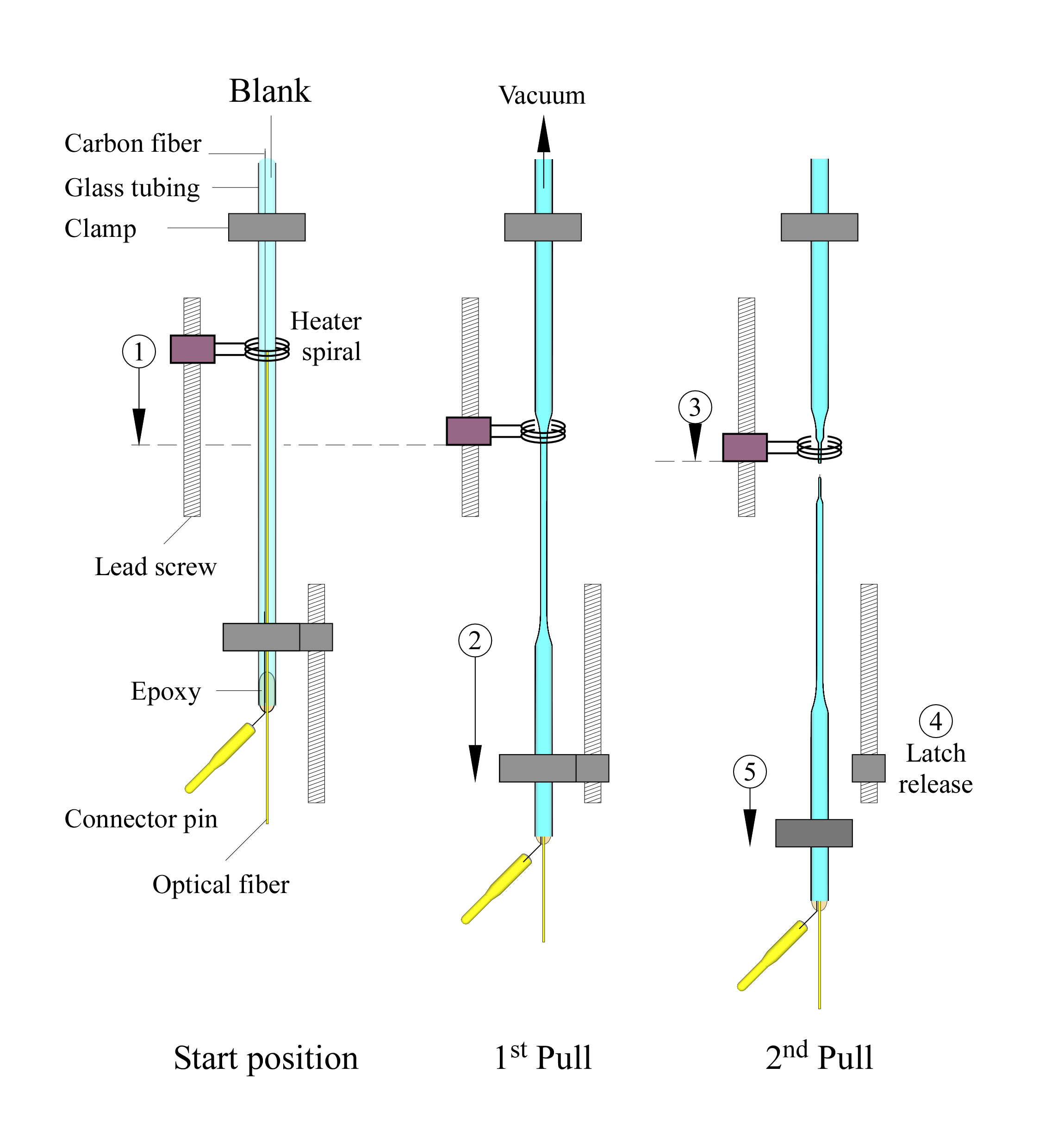

Supplement: S1 Fig — Blanks were made in 1.5 mm diameter borosilicate glass tubes containing optical and carbon fibers. The carbon fiber ended in a gold-plated pin for electrical connection. After vertically fixing the blank in the upper and lower clamps, the heater spiral was positioned so that the end of optical fiber was level with the upper edge of the spiral. Then the spiral was moved down by 15 mm. Once the glass was softened by electrical heating of the spiral, a vacuum was applied and the lower clamp was moved down by 25 mm at a speed of 2 mm/s. Following a 15 s cooling period, the heater spiral was positioned down by 3 mm; the clamp and the movement mechanism were disconnected using a latching device. Lastly, the glass was softened again with electric heating and the final tip was formed by free fall of the lower clamp. All other movements were actuated by lead screws connected to stepper motors as well as timing of events and applying heater currents were programmably executed using a computerized system. (TIF) [file pone.0193836.s001.tif]

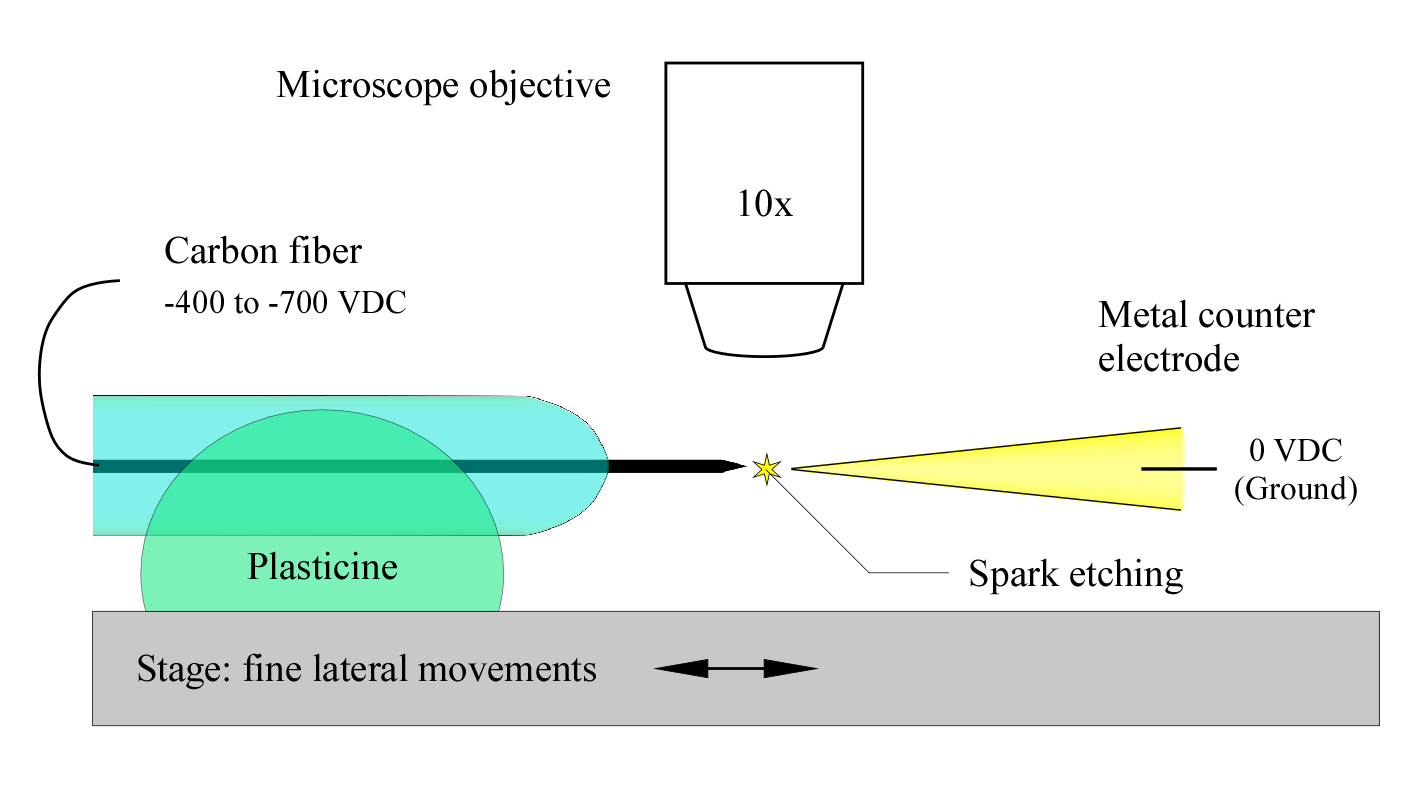

Supplement: S2 Fig — Carbon tip lengths were finalized under a light microscope using spark etching as shown. High voltage electric discharges were generated between the carbon fiber and a sharp tungsten counter microelectrode resulting in a sharply pointed carbon tip. (TIF) [file pone.0193836.s002.tif]

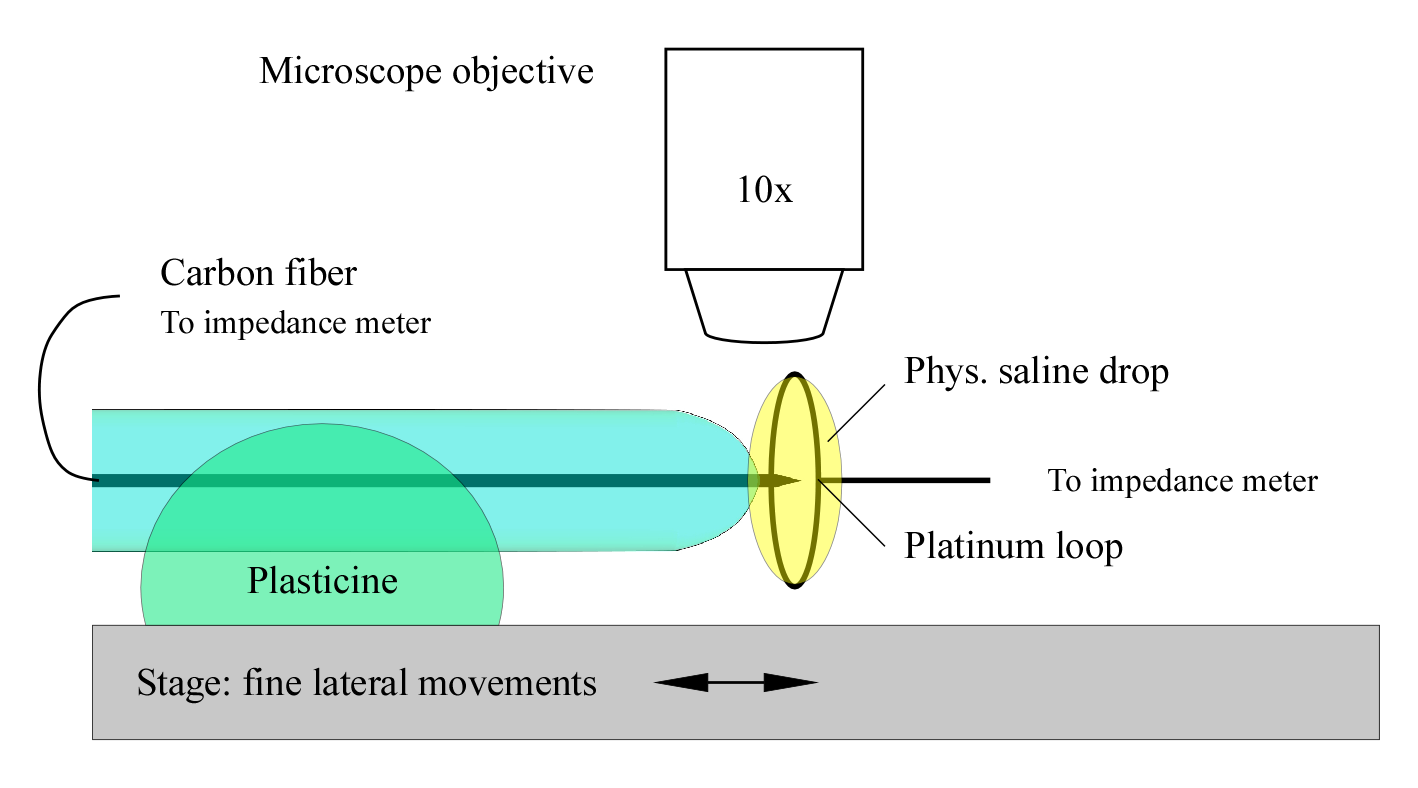

Supplement: S3 Fig — Impedance of micromanufactured carbon tips was determined under a light microscope in a drop of applied physiological saline. Occurring at the same time, tips were visually inspected for imperfections and checked for possible inward liquid suction by capillary forces between the carbon fiber and glass support. (TIF) [file pone.0193836.s003.tif]

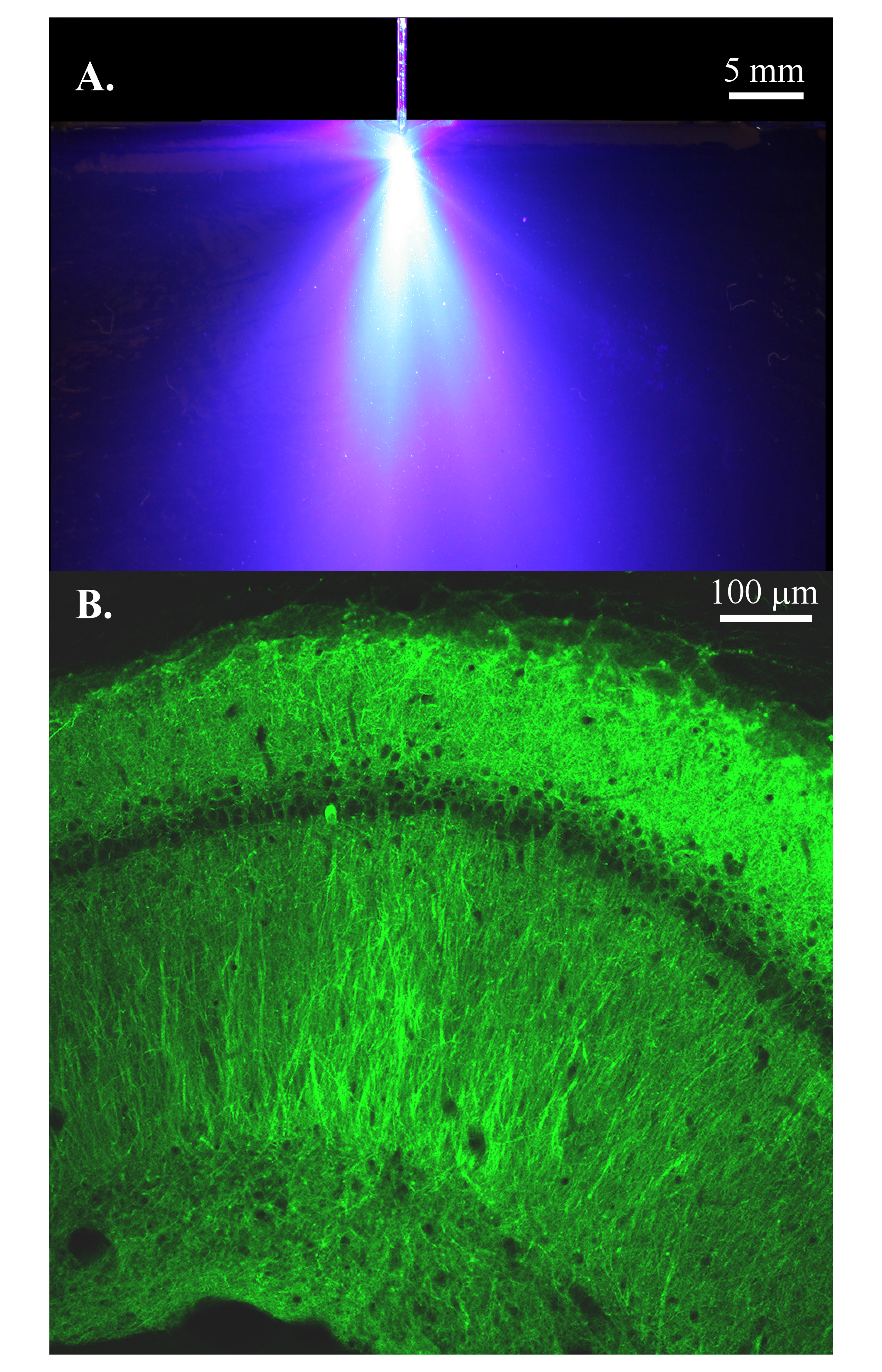

Supplement: S4 Fig — (A) The scattering of light leaving a dome-shaped tip was visualized in agar-agar gel. The borosilicate glass taper extended beyond the edge of the integrated optical fiber and served as a light guide. The half angle of most of the forward light projection was estimated at about 36°. (B) Histological section from the hippocampal region of the rat brain displaying the channelrhodopsin-2 expression. The in vivo recordings were taken from the same area. (TIF) [file pone.0193836.s004.tif]
